# Ultrasound non-invasive measurement of intracranial pressure in neurointensive care: A prospective observational study

**DOI:** 10.1371/journal.pmed.1002356

**Published:** 2017-07-25

**Authors:** Chiara Robba, Danilo Cardim, Tamara Tajsic, Justine Pietersen, Michael Bulman, Joseph Donnelly, Andrea Lavinio, Arun Gupta, David K. Menon, Peter J. A. Hutchinson, Marek Czosnyka

**Affiliations:** 1 Neurosciences Critical Care Unit, Addenbrooke’s Hospital, University of Cambridge, Cambridge, United Kingdom; 2 Department of Neuroscience, University of Genoa, Genoa, Italy; 3 Brain Physics Laboratory, Division of Neurosurgery, Department of Clinical Neurosciences, Addenbrooke’s Hospital, University of Cambridge, Cambridge, United Kingdom; 4 Division of Neurosurgery, Department of Clinical Neurosciences, Addenbrooke’s Hospital, University of Cambridge, Cambridge, United Kingdom; 5 Department of Anaesthesia, Addenbrooke’s Hospital, University of Cambridge, Cambridge, United Kingdom; 6 Division of Neurosurgery, University of Cambridge Hospitals, Cambridge, United Kingdom; 7 Institute of Electronic Systems, Warsaw University of Technology, Warsaw, Poland; Oregon Health and Science University, UNITED STATES

## Abstract

**Background:**

The invasive nature of the current methods for monitoring of intracranial pressure (ICP) has prevented their use in many clinical situations. Several attempts have been made to develop methods to monitor ICP non-invasively. The aim of this study is to assess the relationship between ultrasound-based non-invasive ICP (nICP) and invasive ICP measurement in neurocritical care patients.

**Methods and findings:**

This was a prospective, single-cohort observational study of patients admitted to a tertiary neurocritical care unit. Patients with brain injury requiring invasive ICP monitoring were considered for inclusion. nICP was assessed using optic nerve sheath diameter (ONSD), venous transcranial Doppler (vTCD) of straight sinus systolic flow velocity (FV_sv_), and methods derived from arterial transcranial Doppler (aTCD) on the middle cerebral artery (MCA): MCA pulsatility index (PI_a_) and an estimator based on diastolic flow velocity (FV_d_). A total of 445 ultrasound examinations from 64 patients performed from 1 January to 1 November 2016 were included. The median age of the patients was 53 years (range 37–64). Median Glasgow Coma Scale at admission was 7 (range 3–14), and median Glasgow Outcome Scale was 3 (range 1–5). The mortality rate was 20%. ONSD and FV_sv_ demonstrated the strongest correlation with ICP (*R* = 0.76 for ONSD versus ICP; *R* = 0.72 for FV_sv_ versus ICP), whereas PI_a_ and the estimator based on FV_d_ did not correlate with ICP significantly. Combining the 2 strongest nICP predictors (ONSD and FV_sv_) resulted in an even stronger correlation with ICP (*R* = 0.80). The ability to detect intracranial hypertension (ICP ≥ 20 mm Hg) was highest for ONSD (area under the curve [AUC] 0.91, 95% CI 0.88–0.95). The combination of ONSD and FV_sv_ methods showed a statistically significant improvement of AUC values compared with the ONSD method alone (0.93, 95% CI 0.90–0.97, *p* = 0.01). Major limitations are the heterogeneity and small number of patients included in this study, the need for specialised training to perform and interpret the ultrasound tests, and the variability in performance among different ultrasound operators.

**Conclusions:**

Of the studied ultrasound nICP methods, ONSD is the best estimator of ICP. The novel combination of ONSD ultrasonography and vTCD of the straight sinus is a promising and easily available technique for identifying critically ill patients with intracranial hypertension.

## Abstract 2

In a observational study, Chiara Robba and colleagues examine the association between ultrasound based non-invasive intracranial pressure and invasive intracranial pressure measurement in neurocritical care patients.

## Author summary

**Why was this study done?:**

- Intracranial pressure (ICP) monitoring is necessary in many clinical scenarios.
- Invasive ICP methods are the gold standard, but have many contraindications. Nevertheless, non-invasive ICP (nICP) measurement is a poorly developed technique.
- We present a study comparing ultrasound-based nICP measurement techniques with the gold standard.

**What did the researchers do and find?:**

- In a cohort of 64 patients with brain injury, we compared invasive ICP measurement with 3 different ultrasound-based measurements of nICP: optic nerve sheath diameter (ONSD) ultrasonography, arterial transcranial doppler (aTCD)–derived methods, and straight sinus systolic flow velocity (FV_sv_).
- We found that both optic nerve sheath diameter ultrasonography (ONSD) and straight sinus systolic flow velocity (FV_sv_) are strongly correlated with invasive ICP. In addition, the combination of these 2 nICP parameters (ONSD and FV_sv_) resulted in stronger correlation with ICP.

**What do these findings mean?:**

- A novel nICP monitoring method based on combined ONSD ultrasonography and venous transcranial Doppler has shown promising results for the measurement of intracranial pressure in patients with brain injury.
- This ultrasound-based method is low-cost, quick, and based on technology widely available.
- Future prospective studies will be needed to validate these results.

## Introduction

Intracranial hypertension is a frequent and harmful complication of brain injury; it is an important contributing factor for secondary brain injury, and its severity and duration have been correlated with a fatal outcome [1,2].

A recent trial comparing an invasive intracranial pressure (ICP) monitoring protocol with a protocol based on imaging and clinical examination found no significant differences in patient outcome [3]. However, the trial has been criticised for being underpowered and for the methodology used to measure and treat ICP. Thus, invasive monitoring and treatment of intracranial hypertension is still widely recommended in the management of severely brain-injured patients despite a paucity of randomized evidence [4].

Invasive ICP monitoring through an intraventricular catheter or intraparenchymal microtransducer continues to be the standard of care after severe traumatic brain injury, and should be performed when indications are met [5]. Because the use of invasive transducers can cause complications including infection or haemorrhage [6–8], reliable non-invasive ICP (nICP) estimation would be helpful, especially in clinical situations where the risk–benefit balance of invasive ICP monitoring is unclear or when ICP monitoring is not immediately available or is even contraindicated [4]. Several non-invasive methods based on transcranial Doppler and optic nerve sheath diameter (ONSD) ultrasound are gaining clinical popularity due to their safety, availability, and reliability [8–13]. At present, the best accuracy for a non-invasive method reported in the literature [14,15] has been demonstrated by 2-depth high-resolution transcranial Doppler insonation of the ophthalmic artery. This method does not need calibration and is based on the measurement of the balance point when the measured parameters of blood flow velocity waveforms in the intracranial segment of the ophthalmic artery (which reflect ICP) are identical to extracranial segments (which are mechanically compressed by an externally applied pressure). Other authors [16,17] have proposed different methods for continuous nICP monitoring based on the waveform analysis of cerebral blood flow velocity from the middle cerebral artery (MCA) and arterial pressure. However, despite these promising results, non-invasive techniques remain of insufficient accuracy and temporal resolution to replace invasive ICP monitoring [18,19].

The aim of this study was to compare the accuracy of different ultrasound-based methods for nICP measurement in patients with severe traumatic brain injury undergoing invasive ICP monitoring. Such methods included the ultrasound measurement of the ONSD, venous transcranial Doppler (vTCD), and derived indices obtained from the straight sinus (such as straight sinus systolic flow velocity [FV_sv_]), and arterial transcranial Doppler (aTCD)–derived indices such as middle cerebral artery (MCA) pulsatility index (PI_a_) and diastolic flow velocity (FV_d_).

## Methods

This is a single-centre, prospective observational study conducted from 1 January 2016 to 1 November 2016. Recruited patients were admitted at the Neurosciences Critical Care Unit, Addenbrooke’s Hospital, Cambridge, UK. The protocol was approved by the research ethics boards at the University of Cambridge (REC 15/lo/1918), and written consent was obtained from all participants’ next of kin. The article is reported as per Strengthening the Reporting of Observational Studies in Epidemiology (STROBE) reporting guidelines (S1 Text). Patients older than 18 years requiring sedation, mechanical ventilation, and ICP monitoring with an admission diagnosis of severe traumatic brain injury, aneurysmal subarachnoid haemorrhage, intraparenchymal haemorrhage, or stroke were considered for inclusion. Exclusion criteria were the following: the absence of an informed consent, a known history of ocular pathology or optic nerve trauma, skull base fracture with a cerebrospinal fluid (CSF) leak, inaccessible ultrasound windows (temporal for aTCD and occipital for vTCD), and clinical or radiological suspicion of cerebral venous thrombosis or vasospasm.

### Patient monitoring

Patients were sedated with a continuous infusion of propofol or midazolam, fentanyl, and, when necessary, the muscle relaxant atracurium besylate. Mechanical ventilation was targeted to maintain adequate oxygenation (SaO_2_ > 90%) and normocapnia (PaCO_2_ = 35–40 mm Hg). Intravenous fluids, vasopressors, and inotropic support (norepinephrine and/or epinephrine) were administered to achieve and maintain an adequate cerebral perfusion pressure (CPP > 60 mm Hg). Clinical management was in accordance to international guidelines [20–22].

Treatment of intracranial hypertension was based on a protocol-driven strategy, which included optimisation of arterial blood pressure (ABP) and volaemia, sedation, and infusion of hyperosmolar fluids according to our institutional guidelines.

After a decision to place an ICP monitoring device (by the neurosurgical and intensive care physician in charge), patients were enrolled in the study. ICP was measured via an intraparenchymal probe (Codman & Shurtleff, Raynham, Massachusetts, US) or a catheter inserted into the brain ventricles and connected to an external pressure transducer and drainage system (Codman, Johnson & Johnson, Raynham, Massachusetts, US).

For each patient, we collected the following characteristics: admission Glasgow Coma Scale (GCS), age, sex, height, weight, comorbidities, mechanism and severity of brain injury, and discharge Glasgow Outcome Scale (GOS). The Rotterdam and Marshall scores as well as the Fisher scale were calculated using the admission computer tomography head scan reports [22].

### Ultrasound measurements

Ultrasound measurement was performed by a selected group of experienced operators (TT, JP, MB) using a standardised insonation technique to reduce inter-operator variability. The operators were blinded to the patient’s admission diagnosis, demographics, baseline characteristics, and clinical and physiological background. Operators were not blinded to the actual ICP, but they were blinded to the final formulae to obtain a nICP estimation from the different measurements. Mean arterial blood pressure, end-tidal carbon dioxide partial pressure (ETCO_2_), MCA flow velocities (diastolic [FV_d_], mean [FV_m_], and systolic [FV_s_]), straight sinus flow velocities (diastolic [FV_dv_], mean [FV_mv_], and systolic [FV_sv_]), and ONSD were recorded twice daily from day 1 to day 5 after ICP insertion. Additional measurements were performed in case of acute increases in ICP (above 20 mm Hg). In cases where ICP mean values changed more than ±2 mm Hg during any of the 3 studies (ONSD ultrasound, vTCD, and aTCD), the measurements were excluded from the analysis.

#### ONSD

Ultrasound examination of the ONSD was performed using a 7.5-MHz linear ultrasound probe (11L4, Xario 200; Toshiba, Zoetermeer, The Netherlands) using the lowest possible acoustic power that could measure the ONSD. The probe was oriented perpendicularly in the vertical plane and at around 30 degrees in the horizontal plane on the closed eyelids of both eyes of individuals in supine position with head elevated to 30 degrees. Ultrasound gel was applied on the surface of each eyelid and the measurements were made in the axial and sagittal planes of the widest diameter visible 3 mm behind the retina in both eyes. The final ONSD value was calculated by averaging 4 measured values, as previously described [23,24].

#### Transcranial Doppler

aTCD was performed bilaterally on the MCA through the temporal window using a traditional 2-MHz transducer (5S2, Xario 20; Toshiba, Zoetermeer, The Netherlands) with head elevated to 30 degrees, as previously described [23,25]. The final values of flow velocities were calculated by averaging the 2 measured values.

vTCD was performed on the straight sinus using a 2-MHz transducer (5S2, Xario 20; Toshiba, Zoetermeer, The Netherlands) through an occipital and transforaminal bone window at a depth of 50 to 80 mm for flow directed toward the probe, as previously described [25].

### Statistical analysis

On the basis of previous reports [25–27], we hypothesized that ICP is linearly associated with ONSD, systolic flow velocity on the straight sinus (FV_sv_), PI_a_, and ABP × (1 − FV_d_/FV_m_), and verified this hypothesis in 64 patients. A multivariable linear regression model was obtained from the relationship among ICP, ONSD, and FV_sv_ to derive an nICP estimator based on the combination of ONSD and FV_sv_ (nICP_ONSD+FVsv_).

Deviations from the initial statistical plan (S2 Text) were based on reviewers’ requests and consisted of inclusion of linear mixed effects model analysis for the determination of the estimation formulas for nICP and exclusion of Bland–Altman analysis.

Statistical analysis of the data was conducted with RStudio software (R version 3.1.2). Initially, multiple measurement points were averaged for each patient; therefore, every patient was represented by one single value for all variables assessed. Then, the correlations between ICP and the variables of interest—ONSD, PI_a_, ABP × (1 − FV_d_/FV_m_), and FV_sv_—were verified using the Pearson correlation coefficient (*R*, with the level of significance set at 0.05).

To provide prediction models for ICP estimation, the relationships between ICP and the correlated variables were expressed as linear mixed effects models (R package *lme4* [28]). As fixed effects, we entered ICP and the non-invasive estimators into the model. As random effects, we had intercepts and slopes for the repeated measurement points for each patient (*N* = 445 measurements). A mixed effects multiple regression between ICP and 2 correlated variables, ONSD and FV_sv_, was also performed. Chi-square (χ^2^) values and *p*-values for the comparison of the models were obtained by likelihood ratio tests of the full model with random intercepts and slopes against the null model with random intercepts only.

The area under the curve (AUC) of the receiver operating characteristic (ROC) curve was calculated to determine the ability of the non-invasive methods to detect raised ICP (using a threshold of 20 mm Hg; *N* = 445 measurements). Moreover, we also performed an analysis to determine the best ONSD and FV_sv_ cutoff values for prediction of ICP ≥ 20 mm Hg. In ROC analysis, these are the values presenting the best sensitivity and specificity for prediction of a given threshold. The predicting ability is considered reasonable when the AUC is higher than 0.7 and strong when the AUC exceeds 0.8 [29]. Statistical differences between ROC curves were verified using the DeLong’s test for 2 correlated ROC curves (R package *pROC* [30]).

An analysis of variance (ANOVA) was performed to verify whether any of the variables assessed were associated with mortality in the patient cohort.

## Results

In all, 80 patients with intracranial pathology requiring invasive ICP monitoring were initially considered for enrolment in this study. Among these, 3 were excluded because of the absence of written consent, 2 because it was not possible to find a temporal window, 6 because the occipital window was inaccessible (cervical collar or patient position), 3 because the straight sinus could not be insonated, and 2 because of ocular lesions that precluded the assessment of ONSD.

A total of 445 recordings from 64 patients (each one including ONSD ultrasound, aTCD, and vTCD) were included in the final analysis. The percentage of measurements presenting ICP ≥ 20 mm Hg was 19.3% (*N* = 86). The characteristics of the patients are shown in Table 1. In Table 2, we present the median (interquartile range [IQR]) values of the variables analysed.

**Table 1:** Baseline characteristics of the patient cohort.

Characteristic	*N* (%) or median (IQR)
**Total number**	64
**Male sex**	49 (76.6%)
**Age (years)**	53 (37–64)
**Height (cm)**	175 (165–180)
**Weight (kg)**	78 (67–87)
**Pathology**	
Traumatic brain injury	45 (70.3%)
Aneurysmal subarachnoid haemorrhage	15 (23.4%)
Intracranial haemorrhage	4 (6.3%)
**Comorbidities**	
Hypertension	7 (10.9%)
Depression	12 (18.7%)
Asthma	6 (9.4%)
Alcohol abuse	23 (35.9%)
Smokers	21 (32.8%)
Previous myocardial infarction	1 (3.1%)
**GCS at admission**	7 (3–14)
**GOS at discharge**	3 (1–5)
**Complications**	
Chest infection	12 (18.7%)
Sepsis	2 (3.1%)
Ventriculitis	1 (1.5%)
Post-traumatic ARDS	2 (3.1%)

ARDS, acute respiratory distress syndrome; GCS, Glasgow Coma Scale; GOS, Glasgow Outcome Scale; IQR, interquartile range.

**Table 2:** Median (IQR) values of the studied parameters.

Parameter	Definition	Median (IQR)
ICP (mm Hg)	Intracranial pressure	10 (5–17)
CPP (mm Hg)	Cerebral perfusion pressure	79 (70–87)
ABP_m_ (mm Hg)	Mean arterial blood pressure	90 (85–100)
PaCO_2_ (mm Hg)	Partial pressure of CO_2_	5.20 (4.90–5.90)
ONSD (mm)	Optic nerve sheath diameter	4.9 (4.2–6.0)
FV_s_ (cm/s)	MCA systolic flow velocity (aTCD)	105 (97–114)
FV_d_ (cm/s)	MCA diastolic flow velocity (aTCD)	48 (42–56)
FV_m_ (cm/s)	MCA mean flow velocity (aTCD)	68.67 (59.67–75.00)
FV_sv_ (cm/s)	Straight sinus systolic flow velocity (vTCD)	30 (22–39)
FV_dv_ (cm/s)	Straight sinus diastolic flow velocity (vTCD)	13 (12–18)
FV_mv_ (cm/s)	Straight sinus mean flow velocity (vTCD)	18.67 (15.67–24.67)
PI_a_	MCA pulsatility index (aTCD)	0.86 (0.67–1.04)

aTCD, arterial transcranial Doppler; IQR, interquartile range; MCA, middle cerebral artery; vTCD, venous transcranial Doppler.

### nICP measurement

The correlation analysis between patients revealed good correlation between ICP and ONSD (*R* = 0.76) and between ICP and FV_sv_ (*R* = 0.72), averaged per patient (*N* = 64)—both were statistically significant (*p* < 0.001) and without any influential outliers. The *p*-values for the correlations between ICP and PI_a_ and ABP × (1 − FV_d_/FV_m_) were both non-significant (*p* = 0.63 and *p* = 0.36, respectively). Thus, the regression formulas adopted in this work considered only ONSD and FV_sv_, and the combination of both in a multiple regression model (Table 3).

**Table 3:** Summary of the linear mixed effects models of ICP and the non-invasive estimators across all measurement points (N = 445).

	Full model			Null model		
	Estimate	95% CI	*p*-Value	Estimate	95% CI	*p*-Value
**nICP**_**ONSD**_						
ONSD	4.90	4.39 to 5.38	<0.001	5.00	4.64 to 5.36	<0.001
Intercept	−13.47	−16.05 to −10.84	—	−13.92	−15.99 to −11.85	—
**nICP**_**FVsv**_						
FV_sv_	0.38	0.29 to 0.47	<0.001	0.34	0.29 to 0.40	<0.001
Intercept	0.0005	−2.87 to 2.83	—	1.16	−0.82 to 3.13	—
**nICP**_**ONSD+FVsv**_						
ONSD	4.23	3.63 to 4.79	<0.001	4.54	4.14 to 4.95	<0.001
FV_sv_	0.14	0.07 to 0.22	<0.001	0.10	0.06 to 0.15	<0.001
Intercept	−14.51	−16.82 to −12.19	—	−14.79	−16.83 to −12.75	—

Full model accounts for random intercepts and slopes; null model accounts for random intercepts only.

FV_sv_, straight sinus systolic flow velocity; nICPONSD, non-invasive intracranial pressure estimator based on optic nerve sheath diameter; nICPFV_sv_, non-invasive intracranial pressure estimator based on straight sinus systolic flow velocity; nICPONSD+FV_sv_, non-invasive intracranial pressure estimator based on a combination of ONSD and FV_sv_; ONSD, optic nerve sheath diameter.

Considering the variability in slopes between patients, full models allowing for random intercepts and slopes were significantly better at fitting the data than null models for a nICP estimator based on FV_sv_ (nICP_FVsv_) and for nICP_ONSD+FVsv_ (χ^2^ = 44.19, *p* < 0.001, and χ^2^ = 40.92, *p* < 0.001, respectively). The inclusion of random slopes in the model describing a nICP estimator based on ONSD (nICP_ONSD_) did not produce a significant difference in comparison to the model with random intercepts only (χ^2^ = 2.41, *p* = 0.30). The formulas of the derived models that best fitted the data are described below:

$$nICP_{ONSD}=5.00\times ONSD-13.92\;\left(mm\;Hg\right)$$ (1)

$$nICP_{FVsv}=0.38\times FV_{sv}+0.0005\;\left(mm\;Hg\right)$$ (2)

$$nICP_{ONSD+FVsv}=4.23\times ONSD+0.14\times FV_{sv}-14.51\;\left(mm\;Hg\right)$$ (3)

### Accuracy of the nICP methods

**Fig 1:**
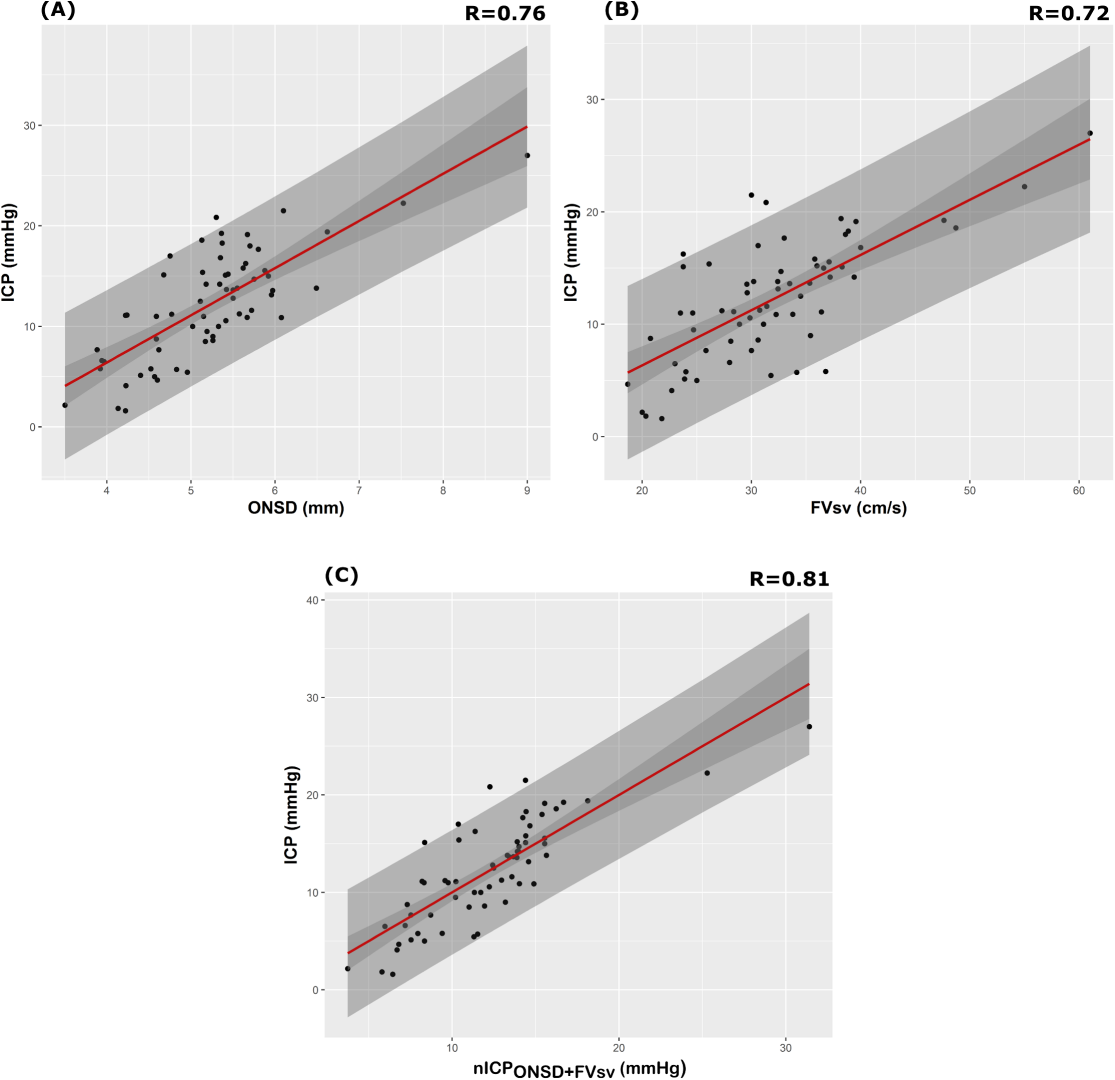
Scatterplot of ICP (mm Hg) and different nICP estimators between patients (N = 64). (A) ONSD method (R = 0.76); (B) FV_sv_ (R = 0.72); (C) nICP estimator based on the combination of ONSD and FV_sv_ (nICPONSD+FV_sv_, R = 0.80). Dark grey shaded areas on the plots represent 95% confidence intervals for the linear regressions; light grey shaded areas on the plots represent the 95% prediction intervals for the linear regressions. ICP, intracranial pressure; nICP, non-invasive intracranial pressure; ONSD, optic nerve sheath diameter.

The correlation coefficient between the ONSD method and ICP averaged per patient (*N* = 64) was *R* = 0.76; the FV_sv_ method showed a correlation with ICP of *R* = 0.72. The combination of the 2 methods presented a correlation coefficient of 0.81 (Table 4; Fig 1). S1 Fig displays the regression plots between ICP and the non-invasive estimators (ONSD and FV_sv_) for each patient, demonstrating the slope variability between patients with multiple measurement points.

**Table 4:** Correlations between ICP and non-invasive estimators across all measurement points (N = 445) and for average values between patients (N = 64).

Estimator	*R* for measurement points (*N* = 445)	*R* for patients (*N* = 64)
ONSD	0.76	0.76
FV_sv_	0.54	0.72
nICP_ONSD+FVsv_	0.78	0.81

FV_sv_, straight sinus systolic flow velocity; nICPONSD+FV_sv_, non-invasive intracranial pressure estimator based on the combination of ONSD and FV_sv_; ONSD, optic nerve sheath diameter; R, Pearson correlation coefficient.

Table 5 summarises the 95% prediction and confidence intervals for the linear regressions between ICP and all non-invasive estimators. The 95% prediction interval for ONSD ranged from 5.05 ± 4.04 to 19.32 ± 4.17 mm Hg; the 95% confidence interval ranged from 11.03 ± 3.95 to 13.34 ± 4.30 mm Hg. The 95% prediction interval for FV_sv_ ranged from 4.57 ± 3.83 to 19.79 ± 3.96 mm Hg; the 95% confidence interval ranged from 10.94 ± 3.71 to 13.43 ± 4.12 mm Hg. For the combination of the 2 methods (nICP_ONSD+FVsv_), the 95% prediction interval ranged from 5.65 ± 4.30 to 18.72 ± 4.45 mm Hg; the 95% confidence interval ranged from 10.90 ± 4.19 to 13.47 ± 4.60 mm Hg.

**Table 5:** Summary of the 95% prediction and confidence intervals (± standard deviations) for the linear regression between intracranial pressure and non-invasive estimators between patients (N = 64).

Estimator	95% prediction interval		95% confidence interval	
	Lower bound	Upper bound	Lower bound	Upper bound
ONSD	5.05 ± 4.04	19.32 ± 4.17	11.03 ± 3.95	13.34 ± 4.30
FV_sv_	4.57 ± 3.83	19.79 ± 3.96	10.94 ± 3.71	13.43 ± 4.12
nICP_ONSD+FVsv_	5.65 ± 4.30	18.72 ± 4.45	10.90 ± 4.19	13.47 ± 4.60

FV_sv_, straight sinus systolic flow velocity; nICPONSD+FV_sv_, non-invasive intracranial pressure estimator based on the combination of ONSD and FV_sv_; ONSD, optic nerve sheath diameter.

**Fig 2:**
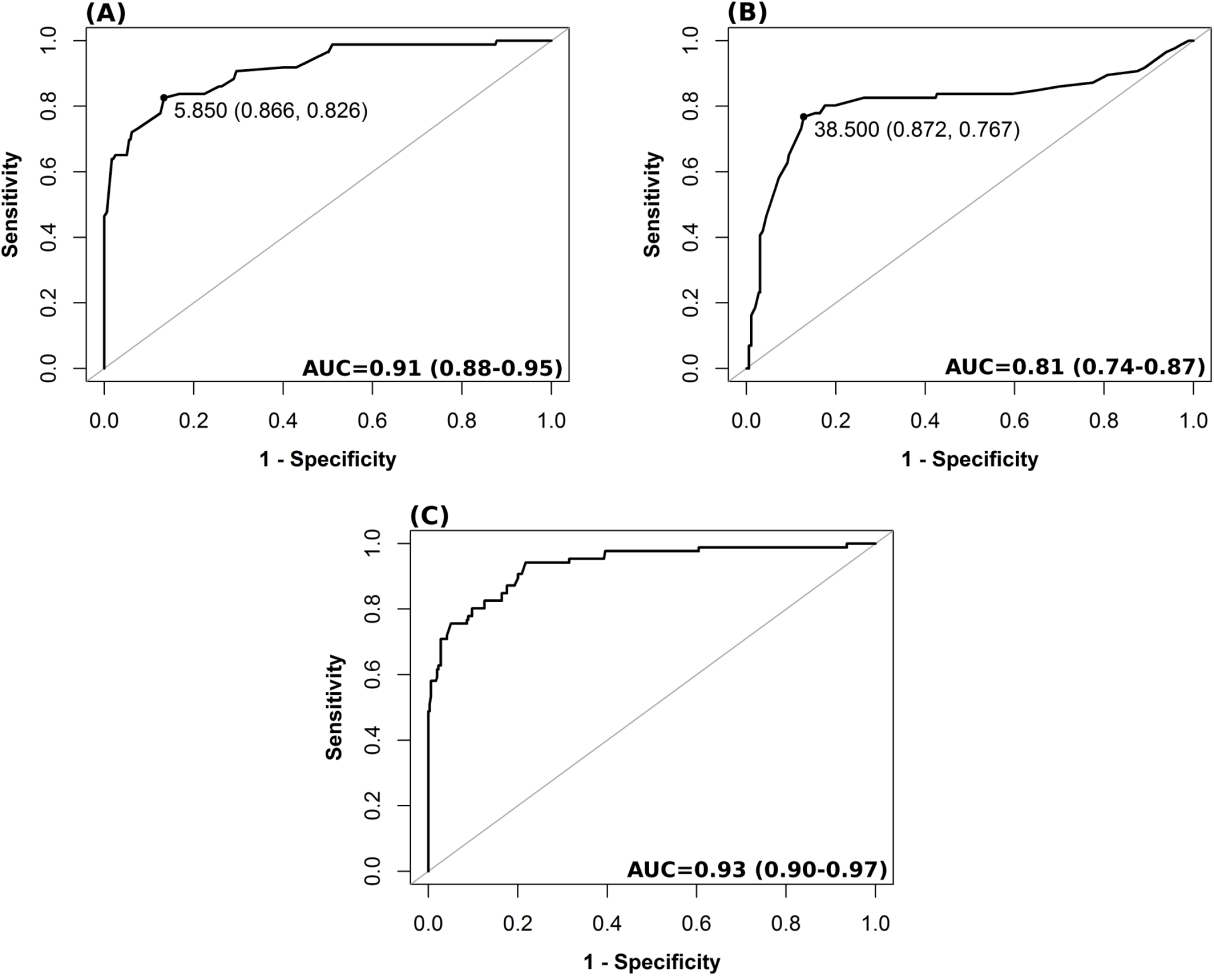
Receiver operating characteristic analysis for different nICP predictors for a threshold of ICP ≥ 20 mm Hg. (A) ONSD method; (B) FV_sv_; (C) nICP estimator based on the combination of ONSD and FV_sv_ (nICPONSD+FV_sv_). The values shown on the curve in (A) and (B) represent the best thresholds (cutoff values presenting the best sensitivity and specificity [in parentheses]) for prediction of intracranial hypertension (ICP ≥ 20 mm Hg), respectively, for ONSD and FV_sv_. AUC is presented followed by the 95% confidence interval. AUC, area under the curve; FV_sv_, straight sinus systolic flow velocity; ICP, intracranial pressure; nICP, non-invasive intracranial pressure; ONSD, optic nerve sheath diameter.

Results of ROC analysis are showed in Table 6 and Fig 2. ONSD had the best AUC among all methods for discriminating cases with intracranial hypertension (ICP ≥ 20 mm Hg) from cases without it (AUC = 0.91, 95% CI 0.88–0.95). The best ONSD and FV_sv_ cutoff values for prediction of intracranial hypertension were 5.85 mm and 38.50 cm/s, respectively. The method based on the combination of ONSD and FV_sv_ showed a statistically significant improvement of AUC values compared with the ONSD method alone (0.93, 95% CI 0.90–0.97, *p* = 0.01 [DeLong’s test]).

**Table 6:** Results of receiver operating characteristic analysis for ICP ≥ 20 mm Hg considering all measurement points (N = 445).

Estimator	AUC (95% CI)
ONSD	0.91 (0.88–0.95)
FV_sv_	0.81 (0.74–0.87)
nICP_ONSD+FVsv_	0.93 (0.90–0.97)

AUC, area under the curve; FV_sv_, straight sinus systolic flow velocity; nICPONSD+FV_sv_, non-invasive intracranial pressure estimator based on the combination of ONSD and FV_sv_; ONSD, optic nerve sheath diameter.

### Mortality and nICP

**Fig 3:**
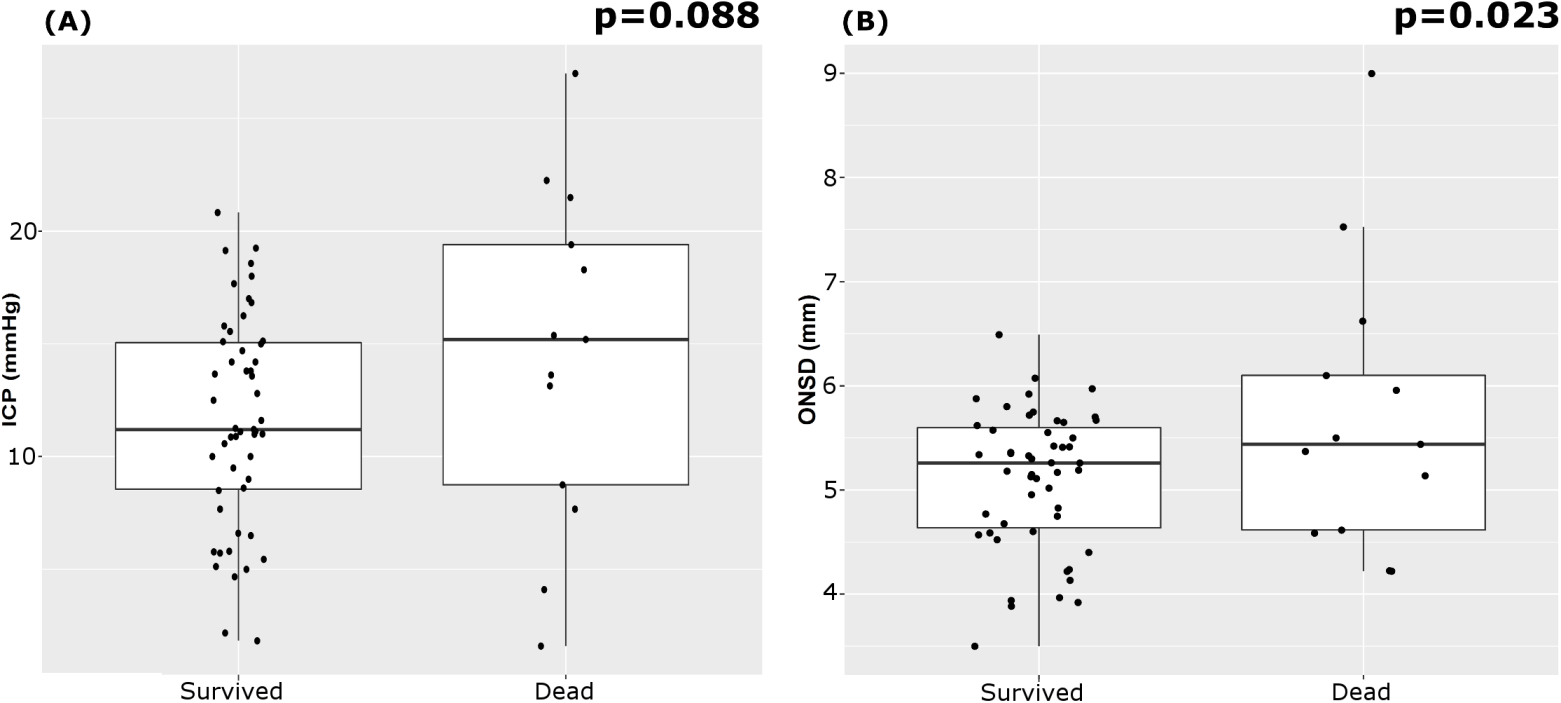
Boxplots of the analysis of variance of ICP and ONSD between patients who survived and those who died. (A) ICP; (B) ONSD. The mean ONSD between patients who survived and died was significantly different. ICP, intracranial pressure; ONSD, optic nerve sheath diameter.

The outcome assessed at discharge revealed that 13 patients died (20%) and 51 survived. Mean ICP showed a tendency to be greater in patients who died; mean ONSD was greater in patients who died than in those who survived (Table 7; Fig 3). FV_sv_ was not significantly different between survivors and non-survivors (*p* = 0.28).

**Table 7:** Summary table describing the association between ICP, ONSD, and mortality.

Measure	Survived (mean ± SD)	Dead (mean ± SD)	Difference	
			Mean	*p*-Value
ICP	11.60 ± 4.65	14.45 ± 7.43	−2.85	0.088
ONSD	5.11 ± 0.66	5.71 ± 1.37	−0.61	0.023

ICP, intracranial pressure; ONSD, optic nerve sheath diameter.

## Discussion

In this study, we present and compare new models for ultrasound-based non-invasive estimation of ICP, based on ONSD ultrasonography, aTCD, and vTCD. Our results show that nICP derived from ONSD has the strongest correlation with invasive ICP. Moreover, ONSD measured through ultrasound was correlated with mortality at discharge. Finally, we demonstrated that a method based on the combination of the 2 best correlated parameters in our cohort (ONSD and FV_sv_—nICP_ONSD+FVsv_) performed even better across all measurement points (*R* = 0.78; AUC for prediction of ICP ≥ 20 mm Hg was 0.93).

Measuring ONSD and FV_sv_ using a duplex Doppler machine is fast and does not require probe fixation or specific dedicated hardware [13]. Furthermore, ultrasonography devices are available in most emergency departments and intensive care units, and are used for many other purposes. Therefore, ultrasonography could be very useful for nICP assessment.

The optic nerve is surrounded by subarachnoid space [10,11,31]; hence, the intraorbital part of the subarachnoid space is distensible and can therefore expand if the CSF pressure increases, with the maximum ONSD fluctuations occurring in the anterior compartment. Although the diameter of the optic nerve is narrower in the anterior than in the posterior segment, increased ICP in the perioptic CSF causes a greater enlargement of the retrobulbar segment of the optic nerve sheath, 3 mm behind the globe, than of the posterior segment [32]. This is probably related to the asymmetrical distribution of the arachnoidal trabeculae and the lower density of the arachnoidal trabeculae in the retrobulbar space. ONSD has been investigated in different clinical scenarios [10,33–35], showing a good correlation with ICP measured invasively and low inter- and intra-observer variability [10,11,27,36]. Our results agree with these findings. Among the studied methods, ONSD was the most accurate in the assessment of ICP; moreover, it is a safe and quick method, as the orbital window is easily available and has no complications.

vTCD for the assessment of ICP is a poorly developed technique. It is known that increasing ICP leads to venous haemodynamic changes, as the part of the cerebral vasculature most sensitive to elevated ICP is the subarachnoid bridging veins. According to the Monro–Kellie doctrine, cerebral compliance strongly depends on the compressibility of the low-pressure venous compartment, and stasis in the pial veins occurs early as a compensatory mechanism in case of increased ICP [37,38]. Consequently, venous blood may be pooled toward larger venous vessels (straight sinus and Rosenthal vein), causing an increase in venous flow velocity. An alternative explanation may be that straight sinus can be compressed by rising ICP, and, with constant volume flow, flow velocity may increase.

Schoser et al. applied vTCD for the estimation of ICP in 30 control volunteers and 25 patients with elevated ICP and found a linear relationship, with strong correlation between mean ICP and FV_sv_ [25]. Similarly to Schoser et al., we found that FV_sv_ is strongly correlated with ICP, whereas other vTCD parameters (venous pulsatility index and FV_dv_) were not good estimators of ICP.

Although the measurement of FV_sv_ seems promising, this technique has some limitations: it can be impossible in polytraumatic patients because of the presence of a cervical collar (6 cases in our cohort). Moreover, the insonation of the straight sinus is feasible in just 72% of cases because of anatomical variations in cerebral veins and transcranial insonation difficulties [25] (even though we had just 3 unsuccessful cases in our cohort, 3.7%).

Our method has several potential clinical applications: it could be useful when invasive monitoring is contraindicated or unavailable, or in many “borderline” situations in which the insertion of invasive monitoring is questioned but a nICP measurement could be useful [20,21]. It can also be applied in patients at risk of intracranial hypertension for causes that are not primarily neurosurgical (such as liver transplantation and intraoperative settings at risk of intracranial hypertension [23,24]) or as screening tool in the emergency department in patients where there is doubt about the need for invasive ICP monitoring.

### Limitations

There are several limitations that deserve to be mentioned. First, transcranial Doppler (and ONSD) measurements were intermittent, and continuous measurements remain more feasible with invasive techniques. Second, the mixed cohort of enrolled patients, including different types of acute brain injury, may represent a bias, as the ICP and cerebral perfusion pressure thresholds for subarachnoid haemorrhage, intracerebral haemorrhage, and stroke are not as well defined as for traumatic brain injury. However, this heterogeneity increases the applicability of the study in many clinical scenarios. Other major limitations are the small number of patients included in this study, the need for specialised training to perform and interpret the ultrasound tests, and the variability in performance among different ultrasound operators.

Finally, most our measurements were obtained in patients with relatively well-controlled ICP. Although a strong correlation between nICP and invasive ICP within the range investigated supports the assumption of validity beyond the range investigated, larger validation studies will need to be performed before non-invasive techniques will be able to substitute for invasive ICP monitoring. In addition, despite our findings showing that mortality has a stronger association with ONSD than with ICP, this does not imply that it would be clinically better to monitor and manage ONSD than ICP.

### Conclusion

A novel nICP monitoring method based on combined ONSD ultrasonography and vTCD was shown to have promising value for the diagnosis of intracranial hypertension, and a strong correlation with invasive ICP monitoring. This ultrasound-based method is quick, low-cost, and based on technology widely available in emergency departments and intensive care units. Whilst we still advocate the superiority of invasive ICP monitoring when this is clearly indicated, the non-invasive methodology presented here may be of potential benefit for ICP assessment in several clinical scenarios where invasive measurement is not immediately available or is contraindicated. However, this method has several limitations, and further studies are needed to confirm and validate our findings.

## Supporting information

S1 Data Database of the patients included in the study. (CSV)

S1 Fig Individual regression plots between ICP and ONSD and ICP and FV_sv_. (A) ICP and ONSD; (B) ICP and FV_sv_. The numbers on the plots represent the patients (*N* = 64), with identities ranked from bottom left (3) to top right (60) on the basis of mean ICP value (in mm Hg). It is noticeable from the individual plots that the association between ICP and each predictor is variable across patients. FV_sv_, straight sinus systolic flow velocity; ICP, intracranial pressure; ONSD, optic nerve sheath diameter.
(PNG)

S1 Text STROBE checklist. (DOC)

S2 Text Protocol of the study and ethical committee form. (PDF)

## Abbreviations

ABP: arterial blood pressure
ARDS: acute respiratory distress syndrome
aTCD: arterial transcranial Doppler
AUC: area under the curve
CPP: cerebral perfusion pressure
CSF: cerebrospinal fluid
FV_d_: diastolic flow velocity
FV_dv_: straight sinus diastolic flow velocity
FV_m_: mean flow velocity
FV_mv_: straight sinus mean flow velocity
FV_s_: systolic flow velocity
FV_sv_: straight sinus systolic flow velocity
GCS: Glasgow Coma Scale
GOS: Glasgow Outcome Scale
ICP: intracranial pressure
IQR: interquartile range
MCA: middle cerebral artery
nICP: non-invasive intracranial pressure
nICP_FVsv_: non-invasive intracranial pressure estimator based on straight sinus systolic flow velocity
nICP_ONSD_: non-invasive intracranial pressure estimator based on optic nerve sheath diameter
nICP_ONSD+FVsv_: non-invasive intracranial pressure estimator based on the combination of optic nerve sheath diameter and straight sinus systolic flow velocity
ONSD: optic nerve sheath diameter
PI_a_: middle cerebral artery pulsatility index
ROC: receiver operating characteristic
vTCD: venous transcranial Doppler
