# Supplementary figures and images for: Ultrasound non-invasive measurement of intracranial pressure in neurointensive care: A prospective observational study
Source: PLoS Med. 2017 Jul 25;14(7):e1002356. doi: 10.1371/journal.pmed.1002356 (PMC5526499; doi:10.1371/journal.pmed.1002356)

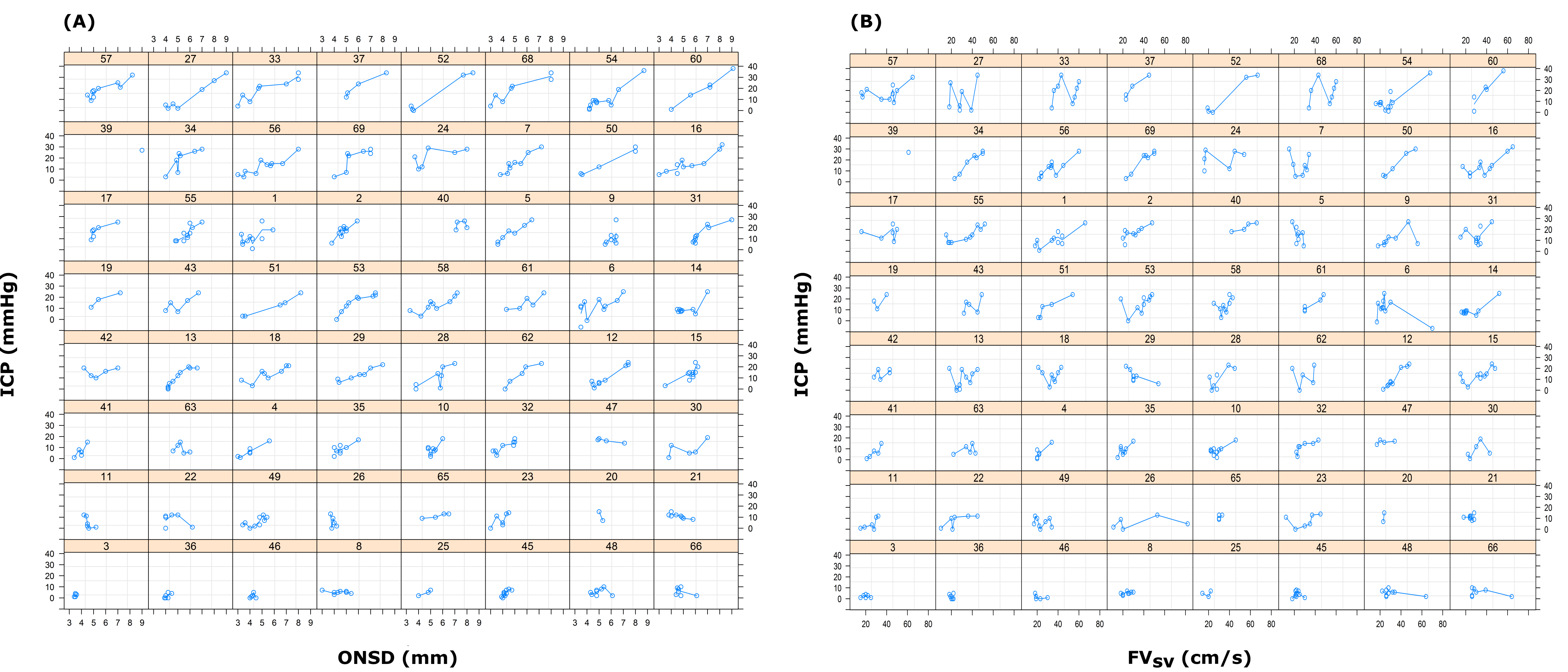

Supplement: S1 Fig — (A) ICP and ONSD; (B) ICP and FVsv. The numbers on the plots represent the patients (N = 64), with identities ranked from bottom left (3) to top right (60) on the basis of mean ICP value (in mm Hg). It is noticeable from the individual plots that the association between ICP and each predictor is variable across patients. FVsv, straight sinus systolic flow velocity; ICP, intracranial pressure; ONSD, optic nerve sheath diameter. (PNG) [file pmed.1002356.s002.png]
