# Supplementary material for: Ultrasound non-invasive measurement of intracranial pressure in neurointensive care: A prospective observational study
Source: PLoS Med. 2017 Jul 25;14(7):e1002356. doi: 10.1371/journal.pmed.1002356 (PMC5526499; doi:10.1371/journal.pmed.1002356)
Supplement: S1 Text — (DOC) [file pmed.1002356.s003.doc]

STROBE Statement—Checklist

|  | Item No | | Recommendation |
| --- | --- | --- | --- |
| **Title and abstract** | 1 | (*a*) Indicate the study’s design with a commonly used term in the title or the abstract  “Ultrasound non-invasive measurement of ICP in neurointensive care: prospective observational study” | |
|  |  | (*b*) Provide in the abstract an informative and balanced summary of what was done and what was found.  Abstract  “**Background:** The invasive nature of the current methods for monitoring of intracranial pressure (ICP) has prevented their use in many clinical situations. Several attempts have been made to develop methods to monitor ICP non-invasively. The aim of this study is to assess the relationship between ultrasound based non-invasive ICP (nICP) and invasive intracranial pressure measurement in neurocritical care patients.  **Methods and Findings:** Prospective observational single cohort study of patients admitted to a tertiary Neurocritical Care Unit with invasive ICP monitoring. Patients with brain injury requiring invasive ICP monitoring were considered for inclusion.  Non-invasive ICP was assessed using: optic nerve sheath diameter (ONSD); venous transcranial Doppler (TCD) systolic velocity (FVsv) in the straight sinus, and middle cerebral artery based nICP estimation using pulsatility index (PIa) and a diastolic velocity derived estimator (FVd).  A total of 445 ultrasound examinations from 64 patients performed from January-November 2016 were included. The median age of the patients was 53 (range 37-64). Median Glasgow Coma Score at admission was 7 (range 3-14), and median Glasgow outcome score was 3 (range 1-5). Mortality rate was 20%.  ONSD and FVsv demonstrated the strongest correlation with ICP (R=0.76 for ONSD vs ICP; R=0.72 for FVsv vs ICP) whereas PIa and the estimator based on FVd did not correlate with ICP significantly. Combining the two strongest nICP predictors (ONSD and FVsv) resulted in an even stronger correlation with ICP (R=0.80). The ability to detect intracranial hypertension (ICP ≥20 mmHg) was highest for ONSD (area under the curve (AUC) 0.91; 95% CI=0.88-0.95) The combination of ONSD and FVsv showed a statistically significant improvement of AUC values compared with the ONSD method alone (0.93; 95% CI=0.90-0.97, p=0.01). Major limitations of this study are the heterogeneity and the small number of patients included in this study, the need for specialized training to perform and interpret the ultrasound tests, and the variability in performance among different ultrasound operators.  **Conclusion:** Of the studied ultrasound nICP methods, ONSD is the best estimator of ICP. The novel combination of ONSD ultrasonography and TCD of the straight sinus is a promising and easily available technique for identifying critically ill patients with intracranial hypertension.” | |
| Introduction | | | |
| Background/rationale | 2 | Explain the scientific background and rationale for the investigation being reported  “Intracranial pressure monitoring is necessary in many clinical scenarios. Invasive methods are the gold standard, but have many contraindications. Non-invasive intracranial pressure measurement is a poorly developed technique” | |
| Objectives | 3 | State specific objectives, including any prespecified hypotheses  “The aim of this study was to compare the accuracy of different ultrasound-based methods for non-invasive ICP measurement in patients with severe traumatic brain injury undergoing invasive ICP monitoring. Such methods included the ultrasound measurement of the optic nerve sheath diameter (ONSD); venous transcranial Doppler (vTCD) and derived indices obtained from the straight sinus (venous flow velocity systolic (FV_sv_)); and arterial TCD (aTCD) derived indices on the middle cerebral artery (MCA) (pulsatility index (PI_a_)) and flow velocity diastolic (FV_d_)).” | |
| Methods | | | |
| Study design | 4 | Present key elements of study design early in the paper:  It is stated in the first paragraph of the method section.  “This is a single-centre, prospective observational study conducted from 1st January 2016 to 1st November 2016. Recruited patients were admitted at the Neurosciences and Trauma Critical Care Unit, Addenbrooke’s Hospital, Cambridge, UK. The protocol was approved by the Research Ethics Boards at the University of Cambridge (REC 15/lo/1918) and written consent was obtained from all participants’ next of kin. Patients older than 18 years old, requiring sedation, mechanical ventilation and ICP monitoring with an admission diagnosis of severe traumatic brain injury, aneurysmal subarachnoid haemorrhage, intraparenchymal haemorrhage and stroke were considered for inclusion. Exclusion criteria were: the absence of an informed consent, a known history of ocular pathology or optic nerve trauma, skull base fracture with a cerebrospinal fluid (CSF) leak, inaccessible ultrasound windows (temporal for aTCD and occipital for vTCD), clinical or radiological suspicion of cerebral venous thrombosis or vasospasm.” | |
| Setting | 5 | Describe the setting, locations, and relevant dates, including periods of recruitment, exposure, follow-up, and data collection  “This is a single-centre, prospective observational study conducted from 1^st^ January 2016 to 1^st^ November 2016. Recruited patients were admitted at the Neurosciences and Trauma Critical Care Unit, Addenbrooke’s Hospital, Cambridge, UK.” | |
| Participants | 6 | Give the eligibility criteria, and the sources and methods of selection of participants. Describe methods of follow-up:  “Patients older than 18 years-old, requiring sedation, mechanical ventilation and ICP monitoring with an admission diagnosis of severe traumatic brain injury, aneurysmal subarachnoid haemorrhage, intraparenchymal haemorrhage and stroke were considered for inclusion. Exclusion criteria were: the absence of an informed consent, a known history of ocular pathology or optic nerve trauma, skull base fracture with a cerebrospinal fluid (CSF) leak, inaccessible ultrasound windows (temporal for aTCD and occipital for vTCD), clinical or radiological suspicion of cerebral venous thrombosis or vasospasm”  No follow-up method applicable. | |
|  |  | (*b*) For matched studies, give matching criteria and number of exposed and unexposed  Not applicable. | |
| Variables | 7 | Clearly define all outcomes, exposures, predictors, potential confounders, and effect modifiers. Give diagnostic criteria, if applicable.  Outcomes are based on the significant correlation between invasively measured ICP and different non-invasive predictors; prediction ability of these estimators in detecting elevated intracranial pressure. No potential confounders or effect modifiers were identified.  Diagnostic criteria not applicable. | |
| Data sources/ measurement | 8* | For each variable of interest, give sources of data and details of methods of assessment (measurement). Describe comparability of assessment methods if there is more than one group. There is a specific section about the methodology:  “Ultrasound measurement was performed by a selected group of experienced operators (TT, JP, MB) using a standardized insonation technique to reduce inter-operator variability. The operators were blinded to the patient’s admission diagnosis, demographics, baseline characteristics, clinical and physiological background. Mean arterial pressure (ABP_m_), end-tidal carbon dioxide partial pressure (ETCO_2_), middle cerebral artery flow velocities (systolic (FV_s_), mean (FV_m_), and diastolic (FV_d_)), straight sinus flow velocities (FV_dv_, FV_mv_, FV_sv_) and ONSD were recorded twice daily from day 1 to 5 post ICP insertion. Additional measurements were performed in case of acute changes in ICP (higher than 20 mmHg). In cases where ICP mean values changed more than ±2 mmHg during any of the three studies (ONSD Ultrasound, vTCD and aTCD), the measurements were excluded from the analysis.  *ONSD*  Ultrasound examination of the ONSD was performed using a 7.5 MHz linear ultrasound probe (11L4, Xario™ 200, Toshiba, Zoetermeer, The Netherlands) using the lowest possible acoustic power that could measure the optic nerve sheath diameter. The probe was oriented perpendicularly in the vertical plane and at around 30 degrees in the horizontal plane on the closed eyelids of both eyes of subjects in supine position. Ultrasound gel was applied on the surface of each eyelid and the measurements were made in the axial and sagittal planes of the widest diameter visible 3 mm behind the retina in both eyes. The final ONSD value was calculated by averaging 4 measured values, as previously described [22,23].  *TCD*  Arterial TCD was performed bilaterally on the MCA through the temporal window using a traditional 2-MHz transducer (5S2 - Toshiba, Xario™ 20, Zoetermeer, The Netherlands) as previously described [22,24].The final values of flow velocities were calculated by averaging the two measured values.  Venous TCD was performed on the straight sinus using a 2-MHz transducer (5S2 - Toshiba, Xario™ 20, Zoetermeer, The Netherlands) through an occipital and transforaminal bone window at a depth of 50 to 80 mmHg for flow directed toward the probe as described by Schoser *et al.* [24].” | |
| Bias | 9 | Describe any efforts to address potential sources of bias.  This is a monocentric study with standardized methods of treatment. Operator was not blinded to actual ICP value, but was blinded to final formula (i.e. what coefficients should be taken to convert jointly ONSD and venous systolic flow velocity to ICP). | |
| Study size | 10 | Explain how the study size was arrived at  A sample size calculation was performed using the Pearson product-moment correlation in which a power of 80%, significance level of 5% and effect size (r=0.3) were considered. This test yielded a sample size of 85 individuals for detecting the specified effect. Based on the number of cases in the area admitted to our Neurosciences Critical Care Unit, we could recruit 80 patients during the study period, with multiple measurements per patient. For the purposes of statistical analysis, we considered 64 patients with 445 measurements in total. | |
| Quantitative variables | 11 | Explain how quantitative variables were handled in the analyses. If applicable, describe which groupings were chosen and why.  “On the basis of previous reports [24–26] we hypothesized that ICP is linearly associated with ONSD, systolic flow velocity on the straight sinus (FV_sv_), PI_a_, ABP*(1-FV_d_/FV_m_), and verified this hypothesis in 64 patients. A multivariable linear regression model was obtained from the relationship among ICP, ONSD and FV_sv_ to derive nICP_ONSD+FVsv_.” | |
| Statistical methods | 12 | (*a*) Describe all statistical methods, including those used to control for confounding | |
|  |  | (*b*) Describe any methods used to examine subgroups and interactions | |
|  |  | (*c*) Explain how missing data were addressed | |
|  |  | (*d*) If applicable, explain how loss to follow-up was addressed | |
|  |  | (*e*) Describe any sensitivity analyses  *(a)*  “Statistical analysis of the data was conducted with R Studio software (R version 3.1.2). Initially, multiple measurement points were averaged for each patient; therefore, every patient was represented by one single value for all variables assessed. Then, the correlations between ICP and the variables of interest were verified: ONSD, PIa, ABP*(1-FVd/FVm) and FVsv using the Pearson correlation coefficient (R, with the level of significance set at 0.05).  Secondly, as to provide prediction models for ICP estimation, the relationships between ICP and the correlated variables were expressed as linear mixed effects models (R package lme4 [28]). As fixed effects, we entered ICP and the non-invasive estimators into the model. As random effects, we had intercepts and slopes for the repeated measurement points for each patient (N=455 measurements). A mixed effects multiple regression between ICP and two correlated variables, ONSD and FVsv, was also performed. Chi-square (χ2) and p-values for comparison of the models were obtained by likelihood ratio tests of the full model with random intercepts and slopes against the null model with random intercepts only.  The area under the curve (AUC) of the receiver operating characteristic curve (ROC) was performed to determine the ability of the non-invasive methods to detect raised ICP (using a threshold of 20 mmHg; N=445 measurements). Moreover, we also performed an analysis to determine the best ONSD and FVsv cut-off values for prediction of ICP ≥20 mmHg. In ROC analysis, these are the values presenting the best sensitivity and specificity for prediction of a given threshold. The predicting ability is considered reasonable when the AUC is higher than 0.7 and strong when the AUC exceeds 0.8 [29]. Statistical differences between ROC curves were verified using the DeLong's test for two correlated ROC curves (R package pROC [30]).  An analysis of variance (ANOVA) was performed to verify whether any of the variables assessed were associated with mortality in the patient cohort.”  *(c)*  The dataset did not contain any missing data for the variables of interest. | |
| Results | | | |
| Participants | 13* | (a) Report numbers of individuals at each stage of study—eg numbers potentially eligible, examined for eligibility, confirmed eligible, included in the study, completing follow-up, and analysed  “80 patients with intracranial pathology requiring invasive ICP monitoring were initially considered for enrolment in this study. A total of 445 recordings from 64 patients (each one including ONSD ultrasound, aTCD, vTCD) were included in the final analysis”. | |
|  |  | (b) Give reasons for non-participation at each stage  Among 80 cases, three were excluded because of the absence of written consent; two because it was not possible to find a temporal window; six because the occipital window was inaccessible (cervical collar or patient position); in three patients, the straight sinus could not be insonated; and finally, two patients were excluded because of ocular lesions which precluded the assessment of ONSD. | |
|  |  | (c) Consider use of a flow diagram:  We believe that the exclusions criteria and selection have been so simple that it doesn’t require a diagram. | |
| Descriptive data | 14* | (a) Give characteristics of study participants (eg demographic, clinical, social) and information on exposures and potential confounders.  Table 1 in the manuscript addresses this issue. | |
|  |  | (b) Indicate number of participants with missing data for each variable of interest:  None. | |
|  |  | (c) Summarise follow-up time (eg, average and total amount)  Not applicable. | |
| Outcome data | 15* | Report numbers of outcome events or summary measures over time.  Not applicable. | |
| Main results | 16 | Give unadjusted estimates and, if applicable, confounder-adjusted estimates and their precision (e.g., 95% confidence interval). Make clear which confounders were adjusted for and why they were included  No confounders were identified. | |
|  |  | (*b*) Report category boundaries when continuous variables were categorized  Not applicable. | |
|  |  | (*c*) If relevant, consider translating estimates of relative risk into absolute risk for a meaningful time period  Not applicable. | |
| Other analyses | 17 | Report other analyses done—eg analyses of subgroups and interactions, and sensitivity analyses  Not applicable. | |
| Discussion | | | |
| Key results | 18 | Summarise key results with reference to study objectives.  This has been done at the beginning of the discussion: “Our results show that, among these methods, nICP derived from ONSD has the strongest correlation with ICP. Moreover, ONSD measured through ultrasound was correlated with mortality at discharge. Finally, we demonstrated that a method based on the combination of the two best correlated parameters in our cohort (ONSD and FV_sv_ - nICP_ONSD+FVsv_) performed even better, (R=0.78 and AUC for prediction of ICP ≥20 mmHg was 0.93)” | |
| Limitations | 19 | Discuss limitations of the study, taking into account sources of potential bias or imprecision. Discuss both direction and magnitude of any potential bias.  We included a limitations sections: “There are several limitations that deserve to be mentioned. Firstly, TCD (and ONSD) measurements were intermittent and continuous measurements remain more feasible with invasive techniques.  Secondly, the mixed cohort of enrolled patients, including different types of acute brain injury, may represent a bias, as the ICP and CPP thresholds for subarachnoid haemorrhage, intracerebral haemorrhage and stroke are not as well defined as for traumatic brain injury. However, this heterogeneity increases the applicability of the study in many clinical scenarios. Other major limitations are the small number of patients included in this study, the need for specialized training to perform and interpret the ultrasound tests, and the variability in performance among different ultrasound operators.  Finally, most our measurements were obtained in patients with relatively well controlled ICP. Although a strong correlation between non-invasive ICP and invasive ICP within the range investigated supports the assumption of validity beyond the range investigated, larger validation studies will need to be performed before non-invasive techniques will be able to substitute invasive ICP monitoring. In addition, despite our findings show there is a stronger association between mortality and ONSD other than ICP, it does not imply that it would be clinically better to monitor and manage ONSD than ICP.” | |
| Interpretation | 20 | Give a cautious overall interpretation of results considering objectives, limitations, multiplicity of analyses, results from similar studies, and other relevant evidence  “ONSD has been investigated in different clinical scenarios [9,32–34] , showing a good correlation with ICP measured invasively and good inter and intra-observed variability [9,10,26,35]. Our results agree with these findings; among the studied methods, ONSD was the most accurate in the assessment of ICP; moreover, it is a safe and quick method as the orbital window is easily available and has no complications.  Venous TCD for the assessment of ICP is a poorly developed technique. It is known that increasing ICP leads to venous haemodynamic changes, as the part of the cerebral vasculature most sensitive to elevated ICP are the subarachnoid bridging veins. According to the Monroe-Kellie doctrine, cerebral compliance strongly depends on the compressibility of the low-pressure venous compartment, and stasis in the pial veins occurs early as a compensatory mechanism in case of increased ICP [36,37]. Consequently, venous blood may be pooled toward larger venous vessels (straight sinus and Rosenthal vein) causing an increase in venous FV. An alternative explanation may be that straight sinus can be compressed by rising ICP, and with constant volume flow, flow velocity may increase.  Venous TCD has been applied for the estimation of ICP in 30 control volunteers and 25 patients with elevated ICP, and the authors found a linear relationship with strong correlation between mean ICP and FVsv in the straight sinus [24]. Similarly to Schoser et al., we found that FVsv is strongly correlated with ICP, whereas other vTCD parameters (PIv and FVdv) were not good estimators of ICP.” | |
| Generalisability | 21 | Discuss the generalisability (external validity) of the study results  In the discussion, we discuss the potential utility of our method in the clinical practice and future applications, and the advocate the role of this method considering the limitations and the need for further validation studies.  “Our method has several potential clinical applications: it could be useful when invasive monitoring is contraindicated or unavailable, or in many “borderline” situations in which the insertion of invasive monitoring is questioned, but a non-invasive ICP measurement could be useful [19,20]. Finally, it can also be applied in patients at risk of intracranial hypertension for non-primarily neurosurgical causes (such as liver transplantation, intraoperative settings at risk of intracranial hypertension [22,23]) or as screening tool in the emergency department in patients where there is doubt about the need for invasive ICP monitoring.” | |
| Other information | | | |
| Funding | 22 | Give the source of funding and the role of the funders for the present study and, if applicable, for the original study on which the present article is based  We had no funding - we attested it in the manuscript. | |

*Give information separately for exposed and unexposed groups.

**Note:** An Explanation and Elaboration article discusses each checklist item and gives methodological background and published examples of transparent reporting. The STROBE checklist is best used in conjunction with this article (freely available on the Web sites of PLoS Medicine at http://www.plosmedicine.org/, Annals of Internal Medicine at http://www.annals.org/, and Epidemiology at http://www.epidem.com/). Information on the STROBE Initiative is available at http://www.strobe-statement.org.
