# Supplementary material for: Ultrasound non-invasive measurement of intracranial pressure in neurointensive care: A prospective observational study
Source: PLoS Med. 2017 Jul 25;14(7):e1002356. doi: 10.1371/journal.pmed.1002356 (PMC5526499; doi:10.1371/journal.pmed.1002356)
Supplement: S2 Text — (PDF) [file pmed.1002356.s004.pdf]

# INVASIVE VS NON-INVASIVE ICP MONITORING BASED ON A COMBINATION OF ONSD AND TCD METHODS IN BRAIN INJURED PATIENTS

OctoberJuly 2015 Version 1

## Study protocol

**TABLE OF CONTENTS**

**1. LIST OF ABBREVIATIONS AND RELEVANT DEFINITIONS........................................4**

**2. SUMMARY.................................................................................................................5**

**3. INTRODUCTION AND RATIONALE...................................................................6**

**3.1 ICP...........................................................................................................................6**

**3.2 Non-invasive system of ICP monitoring...............................................................7**

**3.3 Transcranial Doppler…………………………………………………………………..8**

**3.4 ONSD…………………………………………………………………………………9**

**3.4 Venous Transcranial Doppler………………………………………………………9**

**4. OBJECTIVES**
**……………………………………………………………………………10**

**5. STUDY DESIGN....................................................................................................11**

**5.1 Number of subjects..................................................................................11**

**5.2 Study duration..........................................................................................11**

**5.3 Study site..................................................................................................11**

**6. STUDY POPULATION..........................................................................................11**

**6.1 Population.................................................................................................11**

**6.2 Inclusion criteria........................................................................................11**

**6.3 Exclusion criteria.......................................................................................11**

**7. METHODS...............................................................................................................12**

**7.1 Study endpoint/parameters..........................................................................12**

**7.2 Study procedure………………………………………………………………12**

**7.2.1 ONSD measurement....................................................................................12**

**7.2.2 Transcranial Doppler measurement.............................................................13**

**7.2.3 Venous TCD.................................................................................................13**

**7.3 Ultrasound safety …......................................................................................13**

**7.4 Data to be collected..........................................................................................13**

**8. STATISTICAL ANALYSIS …..........................................................................14**

**9. ETHICAL CONSIDERATIONS…....................................................................15**

**9.1**
**Consent……………………………………………..…………..………………………..15**

**9.2 Declaration of Helsinki…………………………………………………….…………15**

**10. REFERENCES…...............................................................................................18**

**11.APPENDIX……………………………………………………………………………16**

**11.1 Formula for estimation of nICP with TCD on MCA……………….……………….16**

**11.2 Formula for estimation of nICP with TCD on SS……………………….………...16**

**11.3 ICP protocol……………………………………………………………….………17**

## 1. LIST OF ABBREVIATIONS AND RELEVANT DEFINITIONS

| | |
|---|---|
| ICP | Intracranial Pressure |
| nICP | Non-invasive Intracranial pressure |
| aTCD | Transcranial Doppler of middle cerebral artery |
| vTCD | Venous Transcranial Doppler |
| MCA | Middle Cerebral Artery |
| SS | Straight Sinus |
| FV | Flow Velocity |
| ONSD | Optic nerve sheath diameter |
| CPP | Cerebral Perfusion Pressure |
| nCPP | Non-invasive Cerebral Perfusion Pressure |

**Scientific lead:**

**Prof. Marek Czosnyka,** Division of Neurosurgery, Department of Clinical Neurosciences, Addenbrooke's Hospital, University of Cambridge, United Kingdom

Principal Investigator: Dr. Chiara Robba, Department of Anesthesia and Intensive Care. Addenbrooke's Hospital, University of Cambridge, United Kingdom

## 2.SUMMARY


### Rationale

Non-invasive measurement of intracranial pressure (ICP) can be invaluable in the management of critically ill patients. Invasive measurement of ICP remains the gold standard and should be performed when clinical indications are met, but it is invasive and increases risk of infection. Non-invasive monitoring ICP is still a poorly-developed technique. This project focuses on comparing and refining methods for non-invasive ICP assessment based on Transcranial Doppler ultrasonography (TCD) and optic nerve sheath diameter (ONSD).

### Objectives

In this project we aim to verify the accuracy and feasibility of current nICP assessment models and to investigate an innovative system of non-invasive monitoring that combines two different techniques in patients with intracranial hypertension.

### Study design

A single centre prospective observational study

### Study population

We will include in this study brain injured patients requiring invasive ICP monitoring (intraparenchymal and intraventricular) aged > 18 yrs.

### Main study parameters/endpoints

We will assess the concordance between invasive ICP measurement and non-invasive ICP estimated with arterial TCD, venous TCD and ONSD.

## 3.INTRODUCTION AND RATIONALE

### 3.1 ICP

In cases of elevated ICP or systemic hypotension, the cerebral perfusion pressure (CPP) is decreased. CPP is calculated by subtracting ICP from the mean artery pressure (MAP), defined as the sum of the diastolic pressure added to a third of the difference between systolic and diastolic pressure. Under normal physiological conditions, cerebral autoregulation maintains a constant flow of blood to the brain when CPP varies, by dilating or constricting cerebral arteries. However, it is proposed that autoregulation is only effective with a CPP between 50 and 120 mmHg in healthy subjects. Pressure above the upper limit of autoregulation will cause cerebral edema and hyperemia [1-2]. Pressures below the limit lead to insufficient blood flow and cerebral ischemia. A brain injury can cause a state of vasomotor paralysis, where autoregulation is impaired and cerebral blood flow is entirely dependent on CPP.

Additionally, elevated ICP can cause herniation with risk of irreversible brain damage and death[3].According to the American Brain Trauma Foundation, ICP monitoring is indicated in all cases of traumatic brain injury with a Glasgow Coma Scale score (GCS) between 3–8 and an abnormal CT scan, (haematomas, contusion, swelling, herniation, or compressed basal cisterns). Patients with a GCS of 3–8, but with a normal CT scan should be monitored if two or more of the following conditions are present: age over 40, uni- or bilateral motor posturing, or systolic blood pressure under 90 mmHg [4].

Assessment of the ICP is crucial in many neurological and neurosurgical patients because clinical signs of elevated ICP, such as headache, altered level of consciousness, and vomiting, are considered to be nonspecific and unreliable predictors of brain damage. Elevated ICP, defined as ICP over 20 mm Hg, is an important cause of secondary brain injury and may be associated with poor outcome. Conditions where ICP monitoring may be of value include a wide range of disorders, including traumatic brain injury (TBI), intracerebral hemorrhage, subarachnoid hemorrhage (SAH), hydrocephalus, benign intracranial hypertension, meningitis, stroke, etc.

The gold standard for continuous ICP monitoring is an intraventricular catheter connected to an external pressure transducer [5]. This method has been shown to be the most accurate and low-cost method available for ICP monitoring, and it can also be used for therapeutic cerebrospinal fluid (CSF) drainage and administration of drugs. However, the procedure is invasive, and it is at times complicated by infection, haemorrhage, malfunction, obstruction, and malpositioning of the catheter

[3]. Infection rates of 1% to 20% have been reported and two relatively new meta-analyses found hemorrhagic complications in 6% to 7% of patients [6-7].

### 3.2 NON INVASIVE METHOD OF ICP MONITORING

Because of the above mentioned drawbacks with the invasive methods, an accurate non-invasive method to measure ICP has long been looked for [8]. Different techniques have been proposed to estimate ICP but none seem accurate enough to be used as a replacement for invasive ICP measuring methods. These include:

- Radiologic Findings: on CT head and MRI have been demonstrated not to be correlated to ICP values [9-10].
- Transcranial Doppler Sonography: TCD has been used for multiple applications, including non-invasive evaluation of ICP using TCD. Different approaches have been made to analyse the relationship between FV derived parameters, arterial blood pressure (ABP) and ICP [9]. Furthermore a few of the mathematical models, derived from electrical or mechanical analogues of the intracranial dynamics, have been developed in recent years to explain the physiologic basis of ICP waves [11-12].These models use cerebral blood flow velocity and arterial blood pressure waveforms as input variables and to form the nICP signal.
- Tympanic membrane displacement (TMD) indirectly measures cochlear fluid pressure, which directly reflects ICP [13].
- Visual-Evoked Potentials (VEP) accurately reflect disturbances of the visual pathways. Disorders affecting their physiological function, including elevated ICP, can be detected as alterations in VEP [14].
- Examination of the Optic Nerve: The optic nerve sheath (ONS) is continuous with the dura mater; as such, the space within the sheath is continuous with the cranial subarachnoid space. When ICP increases, the pressure in the ONS increases linearly, which distends the ONS. The increased pressure results in stasis of axoplasmic transport which is thought to cause papilloedema. Several methods that make use of changes in the optic nerve can estimate ICP: Scanning Laser Tomography, Magnetic Resonance Imaging, Computer Tomography and ultrasound of the ONS. Several studies have directly correlated ONSD measurements on ultrasound with ICP measured invasively. The cut-off value for normal ONSD, measured 3 mm posterior to the globe, ranges

from 5.2 to 5.9 mm. The sensitivity is 74–95% and the specificity is 74–100% to identify ICP >20 mmHg [15-16].

### 3.3 Transcranial Doppler

TCD measures the velocity of blood flow through the major intracranial vessels by using a low-frequency–pulsed Doppler of 2 MHz over the acoustic window regions where the skull bone is thin [11-12]. Exploiting the Doppler effect, the speed of the blood is assessed by measuring the frequency shift between the incident and reflected wave. TCD generates a velocity–time waveform of cerebral blood flow from which the peak systolic (PSV) and end-diastolic (EDV) flow rates can be measured. Because it is non-invasive, TCD has been used for multiple applications, including detection of changes in cerebral blood flow, vasospasm and circulatory arrest [20-22]. The non-invasive evaluation of ICP using TCD has also been studied. TCD generates a velocity–time waveform of cerebral blood flow from which the peak systolic (PSV) and end-diastolic (EDV) flow rates can be measured. The mean flow velocity (MFV), resistance index (RI), and the pulsatility index (PI) or Gosling index are commonly reported derivations from the waveform display [20-21].

MFV = [(PSV + (EDV 9 2))/3],

RI = (PSV - EDV)/PSV)],

PI = (PSV - EDV)/MFV.

The Gosling pulsatility index (PI), has been for many years the most commonly used index for estimating ICP, but many studies have demonstrated that PI cannot determine the corresponding ICP with an acceptable clinical precision [8,11,12]. In addition, several other formulas and mathematical approaches have been proposed for ICP and CPP estimation [21].

Aaslid et al. [11] proposed a formula for the estimation of CPP based on the relationship between the first harmonic component of the arterial pulse (A1) and the “spectral pulsatility index” (SPI), where SPI is obtained by the first harmonic component of Flow Velocity pulsation, (FV1) divided by the mean Flow Velocity (mFV).

nCPP = A1×mFV/F1

This formula has been demonstrated to be quite sensitive to evaluate the variation of CPP, but it seems to have limited accuracy (predictive error 95% ± 27 mmHg).

Schmitd, Czosnyka et al. [23] proposed a non PI-related formula for estimation of CPP - and therefore ICP: CPP = ABP×FVd/FVm+14 and proved that the absolute difference between real CPP and nCPP so calculated was less than 10 mm Hg in 89% of measurements and less than 13 mm Hg in 92% of measurements in their study. The same group of authors, in another study, (24) reinforced the results of the above mentioned study (correlation between and CPP and measured CPP was r = 0.73; p< 0,0001.

### 3.4 Examination of the Optic Nerve:

The optic nerve sheath (ONS) is continuous with dura mater; as such, the space within the sheath is continuous with the cranial subarachnoid space. When ICP increases, the pressure in the ONS increases linearly, which distends the ONS. [15-18]

The increased pressure results in stasis of axoplasmic transport which is thought to cause papilloedema. Several methods that make use of changes in the optic nerve can estimate ICP: Scanning Laser Tomography, Magnetic Resonance Imaging, Computer Tomography and ultrasound of the ONS. Several studies have directly correlated ONSD measurements on ultrasound with ICP measured invasively. Interestingly, the optic nerve sheath diameter (ONSD) was reported to be increased in patients with intracranial hypertension. Previous reports have indicated that direct measurement of the ONSD is possible by optic nerve sonography and may be applied in brain-injured patients to detect elevated ICP. Although it is difficult to suggest a precise cut-off value, some authors have suggested that the upper normal value of ONSD ranges between 4.5 and 5 mm. The sensitivity is 74–95% and the specificity is 74–100% to identify ICP >20 mmHg [16-18].

### 3.5 Venous Transcranial Doppler

According to the Monroe-Kellie-Cushing-Retzius doctrine, volume increments in any single compartment of the skull, for example, the cerebrospinal fluid (CSF), tissue, and blood compartments, create a pressure gradient involving the other compartments [18]. It is presumed that raised ICP increases cerebrovascular resistance and thereby ultimately restricts cerebral blood flow (CBF). Moreover, the pressure-buffering effect of CSF shifts is considered to be more effective than the vascular system, especially at ICP levels lower than 10 mm Hg. Within a closed skull, cerebral compliance depends secondarily on the compressibility of the low-pressure venous or capacitance segment of the vascular bed. This venous capacitance segment encompasses 70% of the complete cerebral vascular volume. Regional venous stasis with no change in blood flow is described in experimental data obtained during the early stages of ICP dysregulation. With progressive increases in ICP, venous blood flow and then arterial blood flow was impaired.

These venous changes were observed at approximately 50 mm Hg of ICP. Venous transcranial Doppler (TCD) ultrasonography is an evolving technique [25]. The main study in this field has been conducted by Schoser B et al. who performed venous TCD on 30 control volunteers and 25 patients with raised ICP [21]. Venous blood flow velocities (BFVs) in the basal vein of Rosenthal showed, within a certain range, a linear relationship between mean ICP and maximal venous BFV ($r = 0.645$; $p < 0.002$). Moreover, a linear relationship was found for maximal venous BFVs in the SS and mean ICP ($r = 0.928$; $p < 0.0003$).

## 4. OBJECTIVES

We aim to validate and evaluate an innovative system of non-invasive monitoring that uses a combination of ONSD and TCD methods. We will verify the nICP monitoring systems accuracy and feasibility in patients with intracranial hypertension admitted to NCCU for different reasons. Used in isolation both methods have limited accuracy.

**Principal research question**

- What is the accuracy and feasibility of a non-invasive system of ICP monitoring that combines ONSD and TCD (arterial and venous) measurements in patients with intracranial hypertension?

**Secondary research question**

- How do different formulas for TCD (MCA and SS) based nICP estimation perform in patients affected by intracranial hypertension?
- Which single nICP methodology is the most accurate (TCD methods vs ONSD) and does their accuracy change throughout the duration of admission (days 1 to 5)

## 5. STUDY DESIGN

This is a prospective observational study that combines ultrasound ONSD and TCD estimators of nICP, and compares them to invasive ICP in patients affected by intracranial hypertension for acute diseases who have invasive monitoring in situ.

**5.1 Number of Subjects**

50 patients, prospectively enrolled

**5.2 Study duration**

1 year

**5.3 Study site**

Neurosciences Critical Care Unit, Addenbrookes Hospital, Cambridge University Hospitals Trust, Hills Road, Cambridge, CB2 0QQ

**6. STUDY POPULATION**

**6.1** We aim to include in this study patients with intracranial hypertension that require the insertion of ICP monitoring.

**6.2Inclusion Criteria**

50 patients, male and female

Aged >18

Patients with Traumatic Brain Injury, SAH, Stroke, Intracranial Haemorrage and have an ICP monitor in situ (intraparenchimal or intraventricular)

**6.3 Exclusion Criteria**

History of optic nerve lesion or previous optic nerve trauma

Skull base fracture with CSF leak

Inaccessible ultrasound window

**7.METHODS**

**7.1 Study endpoint/parameters**

Prospectively, 50 patients affected by intracranial hypertension for acute diseases (TBI, SAH, Traumatic brain injury, subarachnoid haemorrhage, stroke) will be considered.

These patients will be monitored with continuous intraparenchymal or intraventricular monitoring of ICP. The main study endpoint is to assess the sensitivity and specificity of an innovative non-invasive system to predict raised ICP.

### 7.2 Study procedure

ONSD measurement with ultrasound will be performed and recorded twice a day from day 1 to day 5 from admission, and during highly dynamic periods of ICP monitoring (e.g. plateau waves). Simultaneous measurement of Doppler velocity in the MCA (Middle Cerebral Artery) and venous TCD in the straight sinus will be performed in all the patients who have an invasive ICP to compare the utility of ONSD+ TCD based nICP to asses ICP.

#### 7.2.1 ONSD measurement

ONSD will be measured 3 mm behind the retina. A single investigator will use a 7.5 MHz linear ultrasound probe oriented perpendicularly in the vertical plane and at around 30 degrees in the horizontal plane on the closed eyelids of both eyes of supine subjects. Ultrasound gel will be applied to the outside of each eyelid and recordings made in the axial and longitudinal planes of the widest diameter visible. Measurements will be performed in the transverse and sagittal planes of both eyes, and the final ONSD value will be calculated by averaging 4 measured values. To determine ONSD, electronic calipers will be used to mark 3 mm perpendicularly behind the retina. The ONSD will be measured at the depth marker at right angles to the optic nerve using the lowest possible acoustic power that could measure ICP.

#### 7.2.2 Transcranial Doppler measurement

TCD will be performed on the MCA through the temporal window using a 2-MHz transducer. The TCD measurements will be routinely performed bilaterally on the middle cerebral artery (MCA).

#### 7.2.3 Venous TCD

Venous TCD will be performed on SS through an occipital and transforaminal bone window at a depth of 50 to 80 mmHg for flow directed toward the probe. A brief valsalva maneuver will be performed for confirmation of venous origin.

**7.3 Ultrasound safety**

Ultrasound is considered a routine procedure with almost no complications. However, while it remains unclear whether there are any long-term effects of the diagnostic ultrasound in use today, scientists do know from laboratory studies that ultrasound at high intensities could create immediate effects at the time of exposure, such as heating, referred to as ultrasound's thermal effect. Ultrasound also creates non-thermal effects, also known as mechanical effects. These non-thermal effects include audible sounds, the movement of cells in liquid, electrical changes in cell membranes, shrinking and expansion of bubbles in liquid, and pressure changes.

Diagnostic ultrasound systems now come with displays that warn the system operator when there may be a risk to the patient from the heat or mechanical effects caused by ultrasound. The system displays numbers that provide crude measures of the risk. The Thermal Index (TI) is an estimate of risk from heat, and the Mechanical Index (MI) is an estimate of risk from the non-thermal effects of ultrasound. Manufacturers began incorporating these displays into ultrasound systems in order to meet the US government's 1991 new regulations allowing them to increase ultrasound system outputs. When the MI is above 0.5 or the TI is above 1.0 the risks of ultrasound should be weighed against the benefits [22-23]. Each measurement will be performed according to the current safety guidelines for ultrasound. In particular, to avoid any risk of a retinal lesion, during the ONSD Ultrasound measurement, the acoustic power will be set at the lowest level possible and the examination of the eye will not last longer than 60 seconds.

**7.5 Data to be collected**

Gender and age; male + years

Height and weight; kg + cm

Traumatic Brain Injury classification (Rotterdam and Marshall scale)

SAH Classification (Fisher score)

GCS at admission

Sedation, and paralysis; if yes, specify with which drugs

ICP protocol stage according to ICP protocol of Addenbrooke's Hospital,Cambridge (Appendix 2)

ICP: mmHg

Arterial Blood gases and in particular $PaCO_2$ at the time of the measurement

Ventilator setting:

Peak and plateau pressures; $cmH_2O$
PEEP; $cmH_2O$
Tidal volume; ml
Respiratory rate
Inspiration to expiration ratio
Inspired oxygen fraction; %
Peripheral oxygen saturation; %
End–tidal fractions of $CO_2$; mmHg
Vasoactive drugs; if yes: specify type and dose

## 8.STATISTICAL ANALYSIS

Agreement between ICP measurement and Doppler and ONSD measurement will be evaluated on the continuous raw scale and after categorization based on common usage threshold for ICP (20 mmHg).
The analysis on the continuous scale will use standard indicators, such as Lin's Concordance Correlation Coefficient, a statistic based Pearson's correlation coefficient, however it includes a bias correction term which takes systematic deviations into account
Bland-Altman plot for agreement followed by linear modelling will also be used to evaluate possible trends in measurement differences.

**Sample size will be defined based on preliminary results after the first 30 patients have been registered, and our statistical target will be calculated with a CI 95%, alpha of 5%, and a two tailed Fisher exact test.**

## 9.ETHICAL CONSIDERATIONS

### 9.1 Consent

As patients will lack capacity to decide to participate in this study, whenever possible, a “consultee” will be identified, and their opinion sort about the potential participant’s wishes and feelings in

relation to the project, and whether he or she would have wanted to take part in the study. A member of the research team will approach the consultee to explain the study and its implications. They will be provided with an information sheet, and given time to read it before having to decide if their friend/relative would wish to take part. If they are willing to provide advice on whether their relative/ friend would want to participate in the study, and agree, they will be required to sign a consultee declaration form approved by the ethics committee. Members of the research team will be available to answer any questions, or concerns that the consultee may have both before recruitment and once they are enrolled in the study. Subjects are free to withdraw, or be withdrawn by their consultee if appropriate, at any point in the study, and they need not state a reason.

## 9.2Declaration of Helsinki

The study will be performed in accordance with the guidelines of the Declaration of Helsinki adopted by the 18$^{th}$ World Medical Assembly in Helsinki, Finland in 1964 and amended by subsequent assemblies in Tokyo, Japan in 1975; Venice, Italy in 1983; Hong Kong in 1989; Somerset West, South Africa in 1996; Edinburgh, Scotland in 2000 and Seoul, South Korea, in October 2008 as well as the applicable laws and regulations currently in force.

## 11.APPENDIX

### 11.1Formula used for nICP estimation for TCD on MCA

PI = (PSV - EDV)/MFV.

nCPP(Aaslid formula) = A1×mFV/F1

nCPP(FV diastolic formula) = ABP×FVd/FVm+14

nICP = MABP – nCPP

### 11.2 Formula used for nICP estimation for TCD on SS

PI=BFV(Venous Blood flow velocities)-minimal BFV/mean BFV

### 11.3 ICP protocol

ICP monitoring as clinically indicated. Primary targets:
**ICP $\leq$ 20 mmHg** and **CPP 50 to 70 mmHg**
If advanced monitoring is available, fine-tune treatment based on multimodality targets:
**PRx < 0.2**; **LPR < 25**; **$PbtO_2 \geq$ 15 mmHg**; **$SjO_2$ > 50%**

**STAGE 1**

- **Sedation:** Propofol 2-5 mg/kg/h, Fentanyl 1-4 μg/kg/h; consider Atracurium 0.5 mg/kg/h
- **Ventilation:** $SpO_2 \geq$ 94%; $PaCO_2$ 4.5-5.0 kPa (33-38 mmHg)
- **Circulation:** correct hypovolaemia; vasopressors titrated to CPP > 50 mmHg
- **Temperature:** $\leq$ 37°C (regular paracetamol +/- automated cooling blanket)
- **Nursing:** 15°-30° head up; avoid venous obstruction
- **Antiepileptics:** EEG to exclude seizures +/- institute or escalate antiepileptic therapy

**ICP > 20 mmHg?** Escalate to **STAGE 2**
consider re-scan +/- SOL evacuation

- **CSF drainage:** open EVD (or consider ventricular catheter insertion)
- **Osmotherapy:** Hypertonic NaCl (up to Na $\simeq$ 160 mmol) or Mannitol (up to 2 g/kg QDS)
- **Mild hypothermia:** Temp $\simeq$ 35°C (monitor daily lipids, CK and ECG if still on Propofol)

**ICP > 20 mmHg?** Escalate to **STAGE 3**
consider re-scan +/- SOL evacuation

- **Moderate hypothermia:** Temp 32°C-34°C (change sedation to Midazolam)
- **Ventilation:** $PaCO_2 \simeq$ 4.0 kPa (30mmHg) if $SjO_2$ > 50% and $PbtO_2$ > 15 mmHg

**ICP > 20 mmHg?** Escalate to **STAGE 4**
consider re-scan +/- SOL evacuation

- **Burst suppression:** Thiopentone 5 mg/kg + 3-8 mg/Kg/h (titrate to EEG S.R. $\simeq$ 50%)
- **Surgical decompression:** bifrontal or large fronto-temporo-parietal craniectomy

## 10.REFERENCES

1.Miller JD, Becker DP, Ward JD, et al. Significance of intracranial hypertension in severe head injury. J Neurosurg. 1977;47:503–516.

2.Czosnyka M, Pickard JD. Monitoring and interpretation of intracranial pressure. J Neurol Neurosurg Psychiatry. 2004;75: 813–821.

3.P. J. Hutchinson, A. G. Kolias, M. Czosnyka, P. J. Kirkpatrick, J. D. Pickard, and D. K. Menon, “Intracranial pressure monitoring in severe traumatic brain injury,” British Medical Journal, vol. 346, Article ID F1000, 2013.

4.Brain Trauma Foundation; American Association of Neurological Surgeons; Congress of Neurological Surgeons; Joint Section on Neurotrauma and Critical Care, AANS/CNS, Bratton SL, Chestnut RM, Ghajar J,etJ Neurotrauma. 2007;24 Suppl 1:S91-5. No abstract available. Erratum in: J Neurotrauma. 2008 Mar;25(3):276-8. multiple author names added.

5.Online TBI guidelines 2010 [online]. Available at: www.braintrauma.org/coma-guidlines.Accessed May 14.

6.Binz DD, Toussaint LGIII, Friendman JA. Hemorragic Complications of ventriculostomy placement: a meta-analysis. Neurocrit Care. 2009;10:253-256.

7.Bauer DF, Razdan SN, Bartolucci AA et al. Meta-analysis of hemorragic complications from ventriculostomy placement by neurosourgeons. Neurosurgery. 2011;69:255-260.

8.Kristiansson H, Nissborg E et al. Measuring Elevated Intracranial Pressure through Noninva- sive Methods: A Review of the Literature. J Neurosurg Anesthesiol 2013;25:372– 385!

9.Rosenberg JB, Shiloh AL, Savel RH, et al. Non-invasive methods of estimating intracranial pressure. Neurocrit Care. 2011;15: 599–608

10. N. J. Alperin, S. H. Lee, F. Loth, P. B. Raksin, and T. Lichtor, “MR-intracranial pressure (ICP): a method to measure intracranial elastance and pressure noninvasively by means of MR ima- ging: baboon and human study,” Radiology, vol. 217, no. 3, pp. 877–885, 2000.

11.Aaslid R, Markwalder TM, Nornes H. Noninvasive transcranial Doppler ultrasound recording of flow velocity in basal cerebral arteries. J Neurosurg. 1982;57:769–774.

12.Gosling RG, King DH. Arterial assessment by Doppler-shift ultrasound. Proc R Soc Med. 1974;67:447–449.

13.S. Shimbles, C. Dodd, K. Banister, A. D. Mendelow, and I. R. Chambers, “Clinical comparisonof tympanic membrane displacement with invasive intracranial pressure measurements, “Physiological Measurement, vol. 26, no. 6, pp. 1085– 1092, 2005

14.Acta Neuror (Wien). 2012 Jan;154(1):121-7. doi: 10.1007/s00701-011-1152-9. Epub 2011 Sep 30. Visual evoked potentials are unreliable as markers of ICP due to high variability in normal subjects

15.Rajajee V, Vanaman M, Fletcher JJ, et al. Optic nerve ultrasound for the detection of rai- sed intracranial pressure. Neurocrit Care. 2011;15:506–515.

16.Moretti R, Pizzi B, Cassini F, et al. Reliability of optic nerve ultrasound for the evalua- tion of patients with spontaneous intracranial hemorrhage. Neurocrit Care. 2009;11:406–410.

17.Launey Y, Nesseler N, Le Maguet P, et al. (2014) Effect of Osmotherapy on Optic Nerve Sheath Diameter in Patients with Increased Intracranial Pressure. J Neurotrauma 31:984–8. doi: 10.1089/neu.2012.2829

18. Helmke K, Hansen HC (1996) Fundamentals of transorbital sonographic evaluation of optic nerve sheath expansion under intracranial hypertension. I. Experimental study. Pediatr Radiol 26:701–5.

19.Kalantari H, Jaiswal R, Bruck I, et al. (2013) Correlation of optic nerve sheath diameter measurements by computed tomography and magnetic resonance imaging. Am J Emerg Med 31:1595–7. doi: 10.1016/j.ajem.2013.07.028 19

20. Zweifel C, Czosnyka M, Carrera E, et al. Reliability of the Blood Flow Velocity Pulsatility Index for assessment of intracranial and cerebral perfusion pressures in head injured patients. Neu- rosurgery. 2012;71:237–239.

21. Bellner J, Romner B, Reinstrup P, et al. Transcranial Doppler sonography pulsatility index (PI) reflects intracranial pressure (ICP). Surg Neurol. 2004;62:45–51; discussion 51.

22. Edouard AR, Vanhille E, Le Moigno S, et al. Non-invasive assessment of cerebral perfusion pressure in brain injured patients with moderate intracranial hypertension. Br J Anaesth. 2005;94: 216–221.

23.E A Schmidt, M Czosnyka, I Gooskens, S K Piechnik, B F Matta, P C Whitfield, J D Pickard. Preliminary experience of the estimation of cerebral perfusion pressure using transcranial Doppler ultrasonography. J Neurol Neurosurg Psychiatry 2001;70:198–204.

24.M Czosnyka, B Matta et al. Cerebral perfusion pressure in head-injured patients: a noninvasive assessment using transcranial Doppler ultrasonography. J Neurosurg 88:802– 808, 199.

25. Schoser BGH, Riemenschneider N, Hansen HC (1999) The impact of reised intracranial pressure on cerebral venous hemodynamics: a prospective venous transcanial Doppler ultrasonography study. J Neurosurg 91: 744-749,1999
